# Association Between Family History and Early-Onset Atrial Fibrillation Across Racial and Ethnic Groups

**DOI:** 10.1001/jamanetworkopen.2018.2497

**Published:** 2018-09-21

**Authors:** Zain Alzahrani, Aylin Ornelas-Loredo, Sara D. Darbar, Abdullah Farooqui, Denise Mol, Brandon Chalazan, N. Elizabeth Villagrana, Mark McCauley, Sorin Lazar, Erik Wissner, Adarsh Bhan, Sreenivas Konda, Dawood Darbar

**Affiliations:** 1 Department of Medicine, University of Illinois at Chicago; 2 Department of Medicine, Jesse Brown VA Medical Center, Chicago, Illinois; 3 Department of Epidemiology and Biostatistics, University of Illinois at Chicago

## Abstract

**Importance:**

There is a genetic predisposition to early-onset atrial fibrillation (EOAF) in European American individuals. However, the role of family history in the pathogenesis of EOAF in racial and ethnic minorities remains unclear.

**Objective:**

To determine whether probands with EOAF across racial and ethnic groups have a higher rate of AF in first-degree family members than racially and ethnically matched control patients with non–early-onset AF (non-EOAF).

**Design, Setting, and Participants:**

In this cohort study, patients prospectively enrolled in a clinical and genetic biorepository were administered baseline questionnaires that included questions about family history of AF. Early-onset AF was defined as AF occurring in probands aged 60 years or younger in the absence of structural heart disease. All other forms were categorized as non-EOAF. Recruitment took place from July 2015 to December 2017. Analysis was performed in January 2018.

**Main Outcomes and Measures:**

Primary analysis of reported family history of AF in first-degree relatives with sensitivity analysis restricted to those in whom a family history was confirmed by medical record review and electrocardiogram.

**Results:**

Of 664 patients enrolled (mean [SD] age, 62 [12] years; 407 [61%] male), 267 (40%) were European American; 258 (39%), African American; and 139 (21%), Hispanic/Latino. There was a family history of AF in 36 probands with EOAF (49%) compared with 128 patients with non-EOAF (22%) (difference, 27%; 95% CI, 14%-40%; *P* < .001). On multivariable analysis, the adjusted odds of a proband with EOAF who was of African descent (odds ratio [OR], 2.69; 95% CI, 1.06-6.91; *P* < .001) or Hispanic descent (OR, 9.25; 95% CI, 2.37-36.23; *P* = .002) having a first-degree relative with AF were greater than those of European descent (OR, 2.51; 95% CI, 1.29-4.87; *P* = .006). Overall, probands with EOAF were more likely to have a first-degree relative with AF compared with patients with non-EOAF (adjusted OR, 3.02; 95% CI, 1.82-4.95; *P* < .001) across the 3 racial and ethnic groups. Atrial fibrillation in a first-degree family member was confirmed in 32% of probands with EOAF vs 11% of those with non-EOAF (difference, 21%; 95% CI, 11%-33%; P < .001). Furthermore, African American (28% vs 5%; difference, 23%; 95% CI, 4%-43%; *P* = .001), European American (35% vs 20%; difference, 15%; 95% CI, 1%-30%; *P* = .03), and Hispanic/Latino (30% vs 5%; difference, 25%; 95% CI, 4%-54%; *P* = .02) probands with EOAF were more likely to have a first-degree relative with confirmed AF vs racially and ethnically matched control patients with non-EOAF. The positive and negative predictive values for a family history of confirmed AF were both 89%.

**Conclusions and Relevance:**

Probands of African or Hispanic/Latino descent with EOAF were more likely to have a first-degree relative with AF when compared with European American individuals. These findings support genetic predisposition to EOAF across all 3 races.

## Key Points

**Question:**

What is the association of family history with the pathogenesis of early-onset atrial fibrillation in patients of African, European, and Hispanic descent?

**Findings:**

In this cohort study of 664 patients, probands with early-onset atrial fibrillation were significantly more likely to have a family history of arrhythmia in first-degree relatives than patients with non–early-onset atrial fibrillation. Compared with European American probands, African American and Hispanic/Latino probands with early-onset atrial fibrillation were more likely to have a first-degree relative with arrhythmia.

**Meaning:**

These findings support genetic predisposition to early-onset atrial fibrillation across individuals of European, African, and Hispanic/Latino descent and have important implications for identifying family members at risk for atrial fibrillation and sequencing candidate genes.

## Teaser

This cohort study compares the prevalence of family history of atrial fibrillation (AF) in patients of European, African, and Hispanic/Latino descent with early-onset AF vs non–early-onset AF.

## Introduction

Over the last 2 decades, linkage analysis, candidate gene, and next-generation sequencing approaches have uncovered mutations in cardiac ion channels, signaling molecules, and myocardial structural proteins linked with familial or early-onset atrial fibrillation (EOAF).^1^ Conversely, genome-wide association studies have identified more than 95 AF risk loci, mostly in white individuals of European descent.^2^ Although these discoveries have provided important insights into the underlying pathophysiology of AF and have identified novel therapeutic pathways, most of the heritability of AF remains unexplained.^3^

Collectively, cohort and epidemiological studies support genetic predisposition toward EOAF in white individuals of European descent.^4,5,6^ While it is firmly established that non-Europeans are at a lower risk for incident AF,^7^ racial and ethnic minorities, especially African American patients, have increased rates of stroke, heart failure, and death compared with white patients.^8^ However, the association of family history and the pathogenesis of EOAF in African American or Hispanic/Latino individuals remains unclear. The aim of this study was to determine whether probands of African, European, and Hispanic descent with EOAF were more likely to have a first-degree relative with arrhythmia when compared with racially and ethnically matched control patients with non-EOAF.

## Methods

### Study Population

The study was performed in patients prospectively enrolled in the University of Illinois at Chicago (UIC) AF Registry, which is made up of a clinical and a genetic registry.^9^ Inclusion criteria were age greater than 18 years with a documented history of AF by electrocardiogram (ECG). Consecutive patients attending the General Cardiology, Arrhythmia, and AF Clinics at UIC Medical Center, Advocate Heart Institute, and the Jesse Brown Veterans Affairs (JBVA) Medical Center were approached to participate in the registry over a 36-month period. Hospitalized patients at UIC and JBVA Medical Centers were also approached for participation in the registry. Written informed consent was obtained from all patients under a protocol approved by the UIC, Advocate Christ, and JBVA institutional review boards. Our report complies fully with the Strengthening the Reporting of Observational Studies in Epidemiology (STROBE) reporting guideline.

### Definitions

The terms used to describe EOAF can be broad and are typically used interchangeably with lone, familial, or idiopathic AF.^10^ We defined EOAF as AF occurring in patients aged 60 years or younger with or without hypertension (HTN) in the absence of structural heart disease by clinical examination, ECG, and echocardiography and AF not associated with cardiac surgery. Hypertension was defined by a history and/or the presence of antihypertensive therapy. Criteria for coronary artery disease included a history of myocardial infarction, previous coronary artery bypass graft surgery, or percutaneous coronary intervention and drug treatment. Congestive heart failure (CHF) was defined by a history of heart failure and/or drug treatment for heart failure. A history of obstructive sleep apnea was considered positive if the patient had positive results from a sleep study as part of routine clinical care or was receiving continuous positive airway pressure therapy. Because obstructive sleep apnea and obesity often coincide as risk factors for AF, we did not include either of these in our criteria for EOAF.

### Material and Data Collection

All study participants enrolled in the UIC AF Registry undergo systematic phenotyping inclusive of detailed medical and drug history, echocardiography, and wearing a 2-week continuous event monitor to assess for asymptomatic episodes of AF. A consistent set of definitions is applied to ensure uniformity of data collection in probands and family members.^11^ Furthermore, patients were asked to complete a questionnaire that included baseline data on age, sex, self-reported race/ethnicity, risk factor status at the time of confirmed diagnosis of AF, and a detailed family history, including focused questions related to AF, stroke, pacemaker implantation, and heart disease in first-degree relatives. The majority of Hispanic/Latino individuals (>90%) in Chicago are of Mexican descent and almost all are white.

### Verification of AF in First-Degree Family Members

Patients who gave a family history of AF, stroke, pacemaker implantation, or heart disease in a first-degree relative on the baseline questionnaire were recontacted after enrollment to verify the diagnosis. Family members who confirmed a family history of AF were enrolled in the registry after providing written informed consent. Medical records of relatives were examined and AF was confirmed by ECG, Holter monitor, or event recorder documentation. We calculated the positive predictive value (PPV) and negative predictive value (NPV) of a family history of AF based on the number of patients in whom AF in first-degree relatives was confirmed.

### Statistical Analysis

Categorical variables are expressed as counts and percentages, and continuous variables are expressed as mean (standard deviation). Bivariate analyses of a continuous and a categorical variable were assessed using 2-sample *t* tests or analysis of variance as appropriate, and 2 categorical variables were compared using the χ^2^ test for independence. Multivariate analysis was performed with logistic regression, and covariates were selected using backward elimination. Left ventricular ejection fraction and race were selected for backward elimination as these were the only 2 significant covariates along with reported and confirmed family history of AF. The selection procedure with this approach initially included all covariates in the model and gave each covariate the opportunity to remain in the model, unlike approaches such as forward addition and stepwise. Interactions between covariates and predictors of family history of AF were assessed using significant change in the regression coefficients. Race was not an effect modifier of family history because the Breslow-Day test for homogeneity of the odds ratios (ORs) resulted in a χ^2^ test statistic of 4.72. Fisher exact tests and intervals for overdispersed binomial outcomes were used to examine statistical differences in proportions of EOAF vs non-EOAF and also the 3 racial and ethnic groups (African American, European American, and Hispanic/Latino). We used SAS statistical software version 9.4 (SAS Institute Inc) for statistical computations. Two-tailed *P* < .05 was considered statistically significant.

## Results

### Baseline Characteristics of Cohort

**Figure 1.**
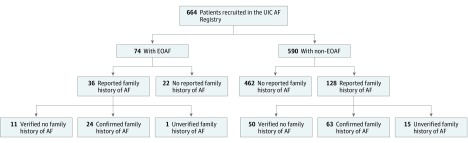
Flowchart Describing the University of Illinois at Chicago (UIC) Atrial Fibrillation (AF) Registry The chart shows the number of patients with early-onset AF (EOAF) and non–early-onset AF (non-EOAF), those reporting a family history of AF, and those in whom the family history was confirmed by electrocardiogram or medical record review.

A flowchart describing the UIC AF Registry cohort, those reporting a family history of AF, and those in whom a family history was verified is shown in Figure 1. We prospectively recruited 664 patients (mean [SD] age, 62 [12] years; 407 [61%] male) with AF in the UIC AF Registry, of whom 267 (40%) were European American, 258 (39%) were African American, and 139 (21%) were Hispanic/Latino. Seventy-four patients (11%) were diagnosed with EOAF and 590 (89%) had non-EOAF. The prevalence of EOAF in African American, European American, and Hispanic/Latino patients was 9% (21 of 245), 15% (43 of 280), and 7% (10 of 139), respectively. Baseline characteristics of the study cohort are shown in Table 1. In our study, 25% of patients reported a family history of AF in a first-degree relative. There was a family history of AF in 36 probands with EOAF (49%) compared with 128 patients with non-EOAF (22%) (difference, 27%; 95% CI, 14%-40%; *P* < .001). As expected, the age at onset was significantly lower in probands with EOAF (mean [SD], 48 [10] years) compared with non-EOAF patients (63 [10] years) (difference, −15%; 95% CI, −17% to −12%; P < .001). All other variables (HTN, obstructive sleep apnea, left ventricular ejection fraction, and left atrial diameter) showed significant differences between the 2 groups except for sex, body mass index (calculated as weight in kilograms divided by height in meters squared), and type of AF.

**Table 1.** Baseline Characteristics of Patients With EOAF and Non-EOAF

Characteristic	No. (%)		Difference (95% CI)	*P* Value for Comparison of EOAF vs Non-EOAF
	EOAF (n = 74)	Non-EOAF (n = 590)		
Age at onset, mean (SD), y	48 (10)	63 (10)	−15 (−17 to −12)	<.001
Male	44 (60)	363 (62)	−0.02 (−0.14 to 0.10)	.73
Ethnicity				
European American	43 (58)	224 (38)	0.20 (0.08 to 0.32)	.003
African American	21 (28)	237 (40)	−0.12 (−0.23 to −0.01)	
Hispanic/Latino	10 (14)	129 (22)	−0.08 (−0.17 to −0.001)	
Family history of AF	36 (49)	128 (22)	0.21 (0.11 to 0.33)	<.001
BMI, mean (SD)	33 (8)	33 (8)	0.5 (−1.6 to 2.5)	.79
Hypertension	26 (35)	505(86)	−0.50 (−0.62 to −0.39)	<.001
Coronary artery disease	0	385 (65)	−0.65 (−0.69 to −0.61)	<.001
Type 2 diabetes mellitus	0	210 (36)	−0.36 (−0.39 to −0.31)	<.001
Congestive heart failure	0	212 (36)	−0.36 (−0.40 to −0.32)	<.001
Structural heart disease	0	127 (22)	−0.22 (−0.25 to −0.18)	<.001
Hyperthyroidism	0	9 (1.5)	−0.01 (−0.03 to −0.01)	<.001
Obstructive sleep apnea	0	119 (20)	−0.20 (−0.23 to −0.17)	<.001
Left ventricular ejection fraction, mean (SD), %	56 (10)	50 (14)	6 (3.9 to 8.9)	<.001
Left atrial diameter, mean (SD), cm	3.9 (1)	4.2 (1)	−0.30 (−0.45 to −0.10)	.002
AF type				
Paroxysmal	55 (70)	445 (76)	−0.04 (−0.15 to 0.06)	.51
Persistent	17 (21)	85 (15)	0.05 (−0.05 to 0.15)	
Permanent	7 (9)	52 (9)	−0.01 (−0.07 to 0.06)	

Abbreviations: AF, atrial fibrillation; BMI, body mass index (calculated as weight in kilograms divided by height in meters squared); EOAF, early-onset AF; non-EOAF, non–early-onset AF.

**Figure 2.**
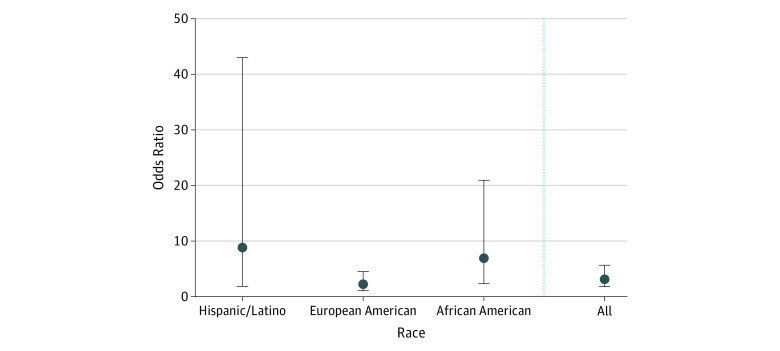
The Odds of a Proband With Early-Onset Atrial Fibrillation Having a Reported Family History of Atrial Fibrillation Compared With Patients With Non–Early-Onset Atrial Fibrillation Across 3 Racial and Ethnic Groups Odds ratios with Wald 95% CIs (error bars) are shown. The dotted blue line separates the odds ratios for each individual race from the 3 races combined.

Baseline characteristics of patients with EOAF and non-EOAF reporting a family history of AF are shown in the eTable in the Supplement. On multivariable analysis, the adjusted odds of a proband who had EOAF and was of African descent (OR, 2.69; 95% CI, 1.06-6.91; *P* < .001) or Hispanic descent (OR, 9.25; 95% CI, 2.37-36.23; *P* = .002) having a first-degree relative with AF were greater than those of a proband with European American descent (OR, 2.51, 95% CI, 1.29-4.87; *P* = .006) (Figure 2). Overall, probands with EOAF were more likely to have a first-degree relative with AF compared with patients with non-EOAF (adjusted OR, 3.02; 95% CI, 1.82-4.95; P < .001) across the 3 racial and ethnic groups.

### Verification of AF in First-Degree Family Members

We were successful in recontacting 148 patients who reported a family history of AF, stroke, pacemaker implantation, or heart disease in a first-degree relative on the initial questionnaire, with 16 patients either declining to contact first-degree relatives or no longer willing to participate in the registry (Figure 1). In 87 patients (24 with EOAF and 63 with non-EOAF), AF was verified by medical record review and ECG documentation in a first-degree relative. Ten first-degree relatives of the 24 patients with EOAF and a confirmed family history were diagnosed with EOAF.

**Figure 3.**
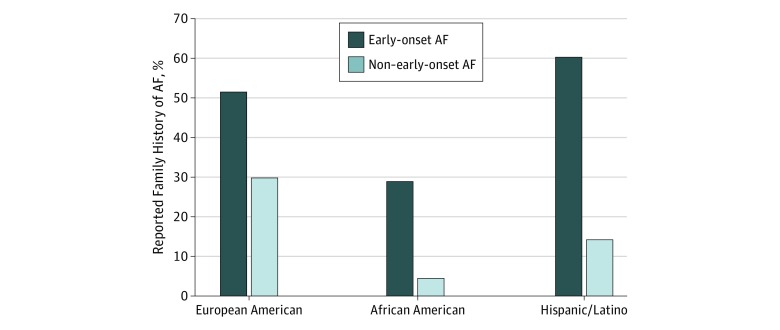
Reported Family History of Atrial Fibrillation (AF) in First-Degree Relatives in Patients With Early-Onset AF and Non–Early-Onset AF Across 3 Racial and Ethnic Groups For European American and Hispanic/Latino groups, *P* < .005 and for African American group, *P* < .001.

The baseline characteristics of patients with EOAF and non-EOAF with or without first-degree relatives with verified AF are shown in Table 2. When comparing patients in the EOAF group with a family history of confirmed AF vs those with no family history of AF, there was no significant difference in age at onset, sex, body mass index, HTN, diabetes mellitus, coronary artery disease, CHF, or left ventricular ejection fraction. In patients with non-EOAF, there were no significant differences in the proportion of first-degree relatives with AF, coronary artery disease, diabetes mellitus, and CHF. A family history of AF in first-degree relatives was confirmed in 24 probands with EOAF (32%) and 63 patients with non-EOAF (11%) (difference, 21%; 95% CI, 11%-33%; *P* < .001). Furthermore, African American (28% vs 5%; difference, 23%; 95% CI, 4%-43%; *P* = .001), European American (35% vs 20%; difference, 15%; 95% CI, 1%-30%; *P* = .03), and Hispanic/Latino (30% vs 5%; difference, 25%; 95% CI, 4%-54%; *P* = .02) probands with EOAF were more likely to have a first-degree relative with confirmed AF when compared with racially and ethnically matched control patients with non-EOAF (Figure 3). On multivariable analysis, the adjusted odds of a proband who had EOAF and was of African (OR, 6.89; 95% CI, 2.30-20.7; *P* < .001) or Hispanic/Latino (OR, 8.78; 95% CI, 1.80-42.7; *P* = .02) descent having a first-degree relative with confirmed AF were greater than those of European descent (OR, 2.19; 95% CI, 1.08-4.45; *P* = .03). Overall, probands with EOAF were more likely to have a first-degree relative with confirmed AF compared with patients with non-EOAF (adjusted OR, 3.16; 95% CI, 1.81-5.55; *P* < .001) across the 3 racial and ethnic groups. Ten first-degree relatives of the 24 probands with EOAF and a confirmed family history were diagnosed with EOAF (10 of 24 [42%]). In contrast, only 7 family members had EOAF in the non-EOAF group (7 of 63 [11%]) (difference, 31%; 95% CI, 6%-54%; *P* = .008). This association supports first-degree family members of probands with EOAF being at increased risk for developing EOAF. The questionnaires for the probands with AF were 100% complete, as they are typically administered verbally and the data verified by review of the medical records. The response rate by first-degree relatives of probands with EOAF was 86%, supporting the likely absence of bias.

**Table 2.** Baseline Characteristics of Patients With Early-Onset Atrial Fibrillation and Non–Early-Onset Atrial Fibrillation Based on a Verified History of Atrial Fibrillation in First-Degree Relatives

Family History	First-Degree Relative With Atrial Fibrillation							
	EOAF (n = 74)				Non-EOAF (n = 590)			
	Yes (n = 24)	No (n = 50)	Difference (95% CI)	*P* Value	Yes (n = 63)	No (n = 527)	Difference (95% CI)	*P* Value
Age at onset, mean (SD), y	46 (11)	50 (10)	−4.7 (−9.4 to −0.77)	.09	61 (10)	63 (11)	−1.9 (−5.0 to 0.9)	.18
Male, No. (%)	13 (54)	31 (62)	−0.08 (−0.32 to 0.16)	.52	35 (56)	328 (62)	−0.06 (−0.20 to 0.06)	.30
Ethnicity, No. (%)								
European American	15 (62)	28 (56)	0.06 (−0.17 to 0.30)	.87	33 (52)	180 (34)	0.35 (9.24 to 0.48)	<.001
African American	6 (25)	15 (30)	−0.05 (−0.26 to 0.16)		21 (34)	221 (42)	−0.22 (−0.33 to −0.11)	
Hispanic/Latino	3 (13)	7 (14)	−0.01 (−0.18 to 0.15)		9 (14)	126 (24)	−0.13 (−0.22 to −0.06)	
BMI, mean (SD)	36 (8.9)	32 (8.0)	4.1 (0.01 to 8.2)	.05	35 (8.4)	33 (8.5)	1.5 (−3.8 to 0.71)	.17
Hypertension, No. (%)	9 (38)	17 (34)	0.04 (−0.20 to 0.27)	.76	52 (83)	453 (86)	−0.03 (−0.13 to 0.06)	.47
Coronary artery disease, No. (%)	0	0	0	>.99	40 (64)	345 (66)	−0.02 (−0.15 to 0.11)	.76
Diabetes mellitus, No. (%)	0	0	0	>.99	19 (30)	191 (36)	−0.06 (−0.18 to 0.06)	.34
Left ventricular ejection fraction, mean (SD), %	56 (10)	56 (10)	−0.6 (−5.4 to 4.3)	.81	52 (13)	50 (15)	2.5 (−1.3 to 6.3)	.19
Congestive heart failure, No. (%)	0	0	0	>.99	23 (63)	189 (36)	0.01 (−0.12 to 0.13)	.92

Abbreviations: BMI, body mass index (calculated as weight in kilograms divided by height in meters squared); EOAF, early-onset atrial fibrillation; non-EOAF, non–early-onset atrial fibrillation.

### Predictive Value of a Family History of AF

We calculated the PPV based on patients who reported a family history of AF in first-degree family members. The PPV of a reported family history of AF was 89% (79 of 89), and the NPV was 89% (67 of 75).

## Discussion

Cumulative evidence from cohort and epidemiological studies supports genetic predisposition toward EOAF in white individuals of European descent. However, the association of family history with the pathogenesis of EOAF across racial and ethnic groups is unclear. We showed that the odds of an African American or Hispanic/Latino proband with EOAF having a first-degree family member with AF were greater than those of European American probands. Overall, probands with EOAF were much more likely to have a first-degree relative with AF compared with patients with non-EOAF across racial and ethnic groups.

Genetic approaches to AF have provided important insights into the underlying genetic mechanisms of AF and identified novel therapeutic pathways.^1^ We identified more than 35 probands with EOAF in whom sequencing of candidate AF genes should be considered.^12^ However, such an approach has a low likelihood (approximately 20%) of identifying an AF-causing variant, and whole exome and genome sequencing may be necessary to identify novel AF genes.^13^ Collectively, the genetic basis of EOAF in racial and ethnic minorities is poorly understood. Despite a greater burden of AF risk factors and worse outcomes,^8,14^ the incidence of AF in African American individuals is substantially less than in European white individuals.^7,15^ While it is presumed that the AF paradox also applies to Hispanic/Latino individuals, we recently confirmed the association between a chromosome 4q25 single nucleotide polymorphism and AF in Mexican American individuals.^9^ In this study, 25% of patients reported a family history of AF in first-degree relatives. This is similar to the percentage reported in the Outcomes Registry for Better Informed Treatment for Atrial Fibrillation (ORBIT-AF) Registry.^16^ However, the lower prevalence of confirmed AF in first-degree family members may in part be due to ascertainment bias, with important differences in the types of patients whose family members can be reached and are willing to participate. Nonetheless, the similar ORs between those with a reported family history of AF and those with confirmed AF and the high PPV and NPV provide reassurance that when a patient reports a family history, it is likely to be accurate.

Our finding that 33% of European American participants with EOAF had a first-degree relative with confirmed AF is consistent with earlier studies reporting a 30% to 40% family history.^6,17^ The adjusted OR of a European American proband with EOAF in our cohort having a first-degree relative with AF (2.51) was similar to that found in the Framingham Heart Study (OR, 3.17).^5^ We also showed that 28% of African American and 60% of Hispanic/Latino probands with EOAF had a confirmed history of AF in first-degree relatives. The upper confidence limit is large for Hispanic/Latino probands with EOAF (Figure 3) because of the lower prevalence of AF among this group. Marcus et al^6^ reported a 7.2-fold greater risk of a first-degree family member having AF in European American individuals. In contrast, we showed a 2.5-fold increased risk of AF in multivariable analyses in white participants, especially if the proband had EOAF. One explanation for these differing results may relate to whether AF in first-degree relatives was verified. Our results are in keeping with those from the Framingham Heart Study.^5^

We showed that family members of probands of African and Hispanic descent with EOAF are at high risk for AF. However, penetrance in familial AF is highly variable.^18,19^ This may relate to the “2-hit” hypothesis for the development of AF, which states that a proband carrying a mutation in a known AF gene only develops the arrhythmia in the presence of a second “hit,” such as HTN or a single-nucleotide polymorphism commonly associated with AF risk.^19,20^ This hypothesis supports performing next-generation sequencing in all patients with EOAF irrespective of AF risk factors. We calculated that the PPV of a first-degree family member having AF was 89%, with incremental increase when probands gave a family history of stroke and pacemaker implantation. The NPV was also high at 89%. Nonetheless, our findings emphasize the importance of obtaining a family history of AF, especially in patients with EOAF.

### Clinical Implications

Our study has a number of important clinical implications. First, our findings emphasize the importance of obtaining a detailed family history in all patients presenting with EOAF irrespective of race or ethnicity. Identifying first-degree family members with AF or at risk for AF is important, as 1 study showed that twins with a co-twin diagnosed with AF had a 20% increased risk of death when compared with twins with an unaffected co-twin.^21^ The increased death rate in co-twins may in part be related to asymptomatic episodes of AF, with clinical and device-based studies reporting that most episodes of AF are asymptomatic.^22,23^ Furthermore, the rates of stroke, CHF, and death are increased in African American and Hispanic/Latino patients with AF compared with white patients of European descent. Thus, first-degree family members of probands with EOAF may warrant closer surveillance monitoring for asymptomatic AF, especially those with established risk factors. Second, given that our data support genetic predisposition to EOAF across races, consideration should be given to sequencing candidate AF genes using next-generation sequencing in probands with EOAF. Although testing for AF genes is not currently recommended,^24^ targeted sequencing in probands with EOAF should be considered as it may affect the management of the proband’s condition and enable cascade screening of first-degree family members who may be at risk for developing AF and adverse outcomes.^8,25^ It is certainly possible that the higher incidence of stroke in ethnic minorities may be related to asymptomatic AF, and identifying family members at higher risk for AF by genetic testing or more intense ECG surveillance is warranted. Furthermore, recent data from the ORBIT-AF Registry suggest that compared with non-Hispanic white patients, Hispanic/Latino patients are more likely to be younger at AF onset, are more likely to be female, and disproportionally experience AF-related stroke.^14^

### Limitations

Our findings that probands with EOAF are more likely to have a first-degree relative with AF when compared with non-EOAF patients across races need to be replicated in diverse independent cohorts. However, AF was not confirmed in all probands with EOAF who reported a family history. This may relate to asking questions not directly related to AF, such as questions about family history of stroke, pacemakers, and heart disease. Although a sensitivity analysis reduced recall bias, some residual bias may remain. We attempted to mitigate confounding clinical factors, eg, asymptomatic AF and language barriers, by providing symptomatic family members with 2-week monitors and Spanish-speaking research coordinators, respectively. To control for confounders such as family size and number of affected family members requires constructing a detailed family pedigree. Such a comprehensive family survey is yet to be performed. Another potential confounder that was not assessed in our observational study is awareness of AF prior to the diagnosis. Although the odds of a first-degree relative developing EOAF are not quantitative, the adjusted OR of a European American proband with EOAF in our cohort having a first-degree relative with AF (2.51; 95% CI, 1.29-4.87) were similar to those found in the Framingham Heart Study (OR, 3.17; 95% CI, 1.71-5.86).^5^ Furthermore, statistically calibrated interpretation of ORs would require comprehensive kindred analyses. Such a survey would also allow us to calculate the recurrence risk ratio for first-degree relatives.

It was not possible to perform simulation studies to assess the impact of false-negative reporting rates on the ORs because of the wide variation of these across EOAF and non-EOAF and races. Furthermore, calculating false-negative rates would be challenging as it would require recontacting approximately 500 patients enrolled in the registry and their first-degree relatives. However, based on the PPV of 89% and NPV of 89%, the misreporting rates are likely to be low. Still, we acknowledge that small changes in the PPV and NPV of false-negative reporting of non-EOAF will affect the estimated ORs. The Hispanic/Latino patients in this cohort are an admixed population, and the prevalence of family history of AF may vary across races and ethnicities within this group. We included HTN in our definition of EOAF, but some studies have excluded patients with this diagnosis. Increasingly, the definition of EOAF is based only on age at onset of AF and is supported by the 2-hit hypothesis. Nonetheless, we acknowledge that HTN itself may be an inherited trait and as such may modify the risk of developing AF. It should be noted that the UIC AF Registry is not a population-based cohort but rather a highly selected cohort of patients recruited from cardiology outpatient clinics and hospitalized patients.

## Conclusions

This study was the first we know of to show that the odds of an African American or Hispanic/Latino proband with EOAF having a first-degree relative with AF were greater than those of probands of European descent. Our findings support genetic predisposition to EOAF across all 3 racial and ethnic groups studied and have important implications for identifying family members at risk for AF and screening candidate genes. The identification of novel AF genes, especially in African American and Hispanic/Latino patients, may not only uncover the underlying molecular mechanisms of AF and identify novel therapeutic pathways, but also provide important insights into the AF paradox across racial and ethnic groups.

## Notes

Supplement. **eTable.** Baseline Characteristics of Patients With EOAF and Non-EOAF Based on a Reported History of AF in First-Degree Relatives
