# Supplementary material for: Association Between Family History and Early-Onset Atrial Fibrillation Across Racial and Ethnic Groups
Source: JAMA Netw Open. 2018 Sep 21;1(5):e182497. doi: 10.1001/jamanetworkopen.2018.2497 (PMC6324458; doi:10.1001/jamanetworkopen.2018.2497)
Supplement: Supplement. — eTable. Baseline Characteristics of Patients With EOAF and Non-EOAF Based on a Reported History of AF in First-Degree Relatives [file jamanetwopen-1-e182497-s001.pdf]

Supplementary Online Content

Alzahrani Z, Ornelas-Loredo A, Darbar SD, et al. Association Between Family History and Early-Onset Atrial Fibrillation Across Racial and Ethnic Groups. *JAMA Netw Open.* 2018;1(5):e182497. doi:10.1001/jamanetworkopen.2018.2497

eTable. Baseline Characteristics of Patients With EOAF and Non-EOAF Based on a Reported History of AF in First-Degree Relatives

This supplementary material has been provided by the authors to give readers additional information about their work.

© 2018 Alzahrani Z et al. *JAMA Network Open.*

eTable. Baseline characteristics of patients with EOAF and non-EOAF based on a reported history of AF in first-degree relatives.

	First degree relative with AF					
	Early-onset AF (n=74)		P-value	Non-early-onset AF (n=590)		P-value
Family History	Yes (n=36)	No (n=38)		Yes (n=128)	No (n=462)	
Age of onset (yrs.)	46±11	51±10	0.06	62±10	64±11	0.13
Male, n (%)	21 (58)	23 (61)	0.85	67 (52)	296 (62)	0.02
Ethnicity			0.46			<.001
European American, n (%)	22 (61)	21 (55)		66 (52)	158 (34)	
African American, n (%)	8 (22)	13 (34)		44 (34)	193(42)	
Hispanic/Latino, n (%)	6 (17)	4 (10)		18 (14)	111(24)	
BMI	35±8.4	31±8.0	0.03	34±7.9	33±8.6	0.15
HTN, n (%)	13 (36)	13 (34)	0.86	109 (85)	396 (86)	0.87
CAD, n (%)	0	0	1.00	89 (64)	296 (64)	0.25
DM, n (%)	0	0	1.00	41 (32)	169 (37)	0.34
LVEF (%)	57±11	56±11	0.54	52±14	49±15	0.11
CHF, n (%)	0	0	1.00	42 (33)	170 (37)	0.41

AF, atrial fibrillation; EO, early-onset; BMI, body mass index; DM, diabetes mellitus; LVEF, left ventricular ejection fraction; CAD, coronary artery disease; CHF, congestive heart failure; HTN, hypertension; OSA, obstructive sleep apnea; SHD, structural heart disease. Mean (SD).

© 2018 Alzahrani Z et al. *JAMA Network Open.*
